# Interacting With a Visiting Dog Increases Fingertip Temperature in Elderly Residents of Nursing Homes

**DOI:** 10.3389/fpsyg.2020.01906

**Published:** 2020-07-31

**Authors:** Anne Nilsson, Linda Handlin, Lena Lidfors, Maria Petersson, Kerstin Uvnäs-Moberg

**Affiliations:** ^1^Department of Animal Environment and Health, Swedish University of Agricultural Sciences, Skara, Sweden; ^2^School of Health Sciences, University of Skövde, Skövde, Sweden; ^3^Department of Molecular Medicine and Surgery, Endocrine and Diabetes Unit, Karolinska Institutet, Stockholm, Sweden

**Keywords:** fingertip temperature, visiting dog, elderly, stress, sympathetic nervous system, relaxation

## Abstract

The aim of this study was to investigate whether interacting with a visiting dog influences fingertip temperature and cortisol levels in residents living in nursing homes for the elderly. The study included two groups, the dog group (*n* = 13) and the control group (*n* = 11–15) and lasted for 8 weeks for the dog group and 6 weeks for the control group. All participants were residents living at nursing homes for the elderly. The researchers visited small groups of the participants twice weekly during the entire study in both the dog and the control group. The visiting dog and the dog handler accompanied the researchers during weeks 3–6. Fingertip temperature was measured and saliva samples for cortisol determination were collected at 0, 20 and 60 min for the dog group and at 0 and 20 min for the control group. For analysis the study was divided into periods; Period 1 (week 1–2), Period 2 (week 3–4), Period 3 (week 5–6) and Period 4 (week 7–8, only the dog group). Mean values based on all data obtained at 0 and 20 min during period 1–3 were compared between groups. A second, separate analysis for the dog group also included data from 60 min and for period 4. For the dog group fingertip temperature increased significantly between period 1 and 2, 1 and 3 and 1 and 4 (*p* < 0.05). In addition, fingertip temperature rose significantly between 0 and 20 min and between 0 and 60 min within all periods. For the control group a significant decrease in fingertip temperature was observed between period 1 and 3 (*p* < 0.05). Fingertip temperature did not differ between the two groups during period 1, but was significantly higher for the dog group than for the control group during periods 2 and 3 (*p* < 0.05 and *p* < 0.001, respectively). Cortisol results are only presented descriptively due to that many samples had too low volume of saliva to be analyzed. In the present study interaction between elderly residents and a visiting dog resulted in increased fingertip temperature, probably reflecting a decrease in the activity of the sympathetic nervous system and therefore a decrease in stress levels.

## Introduction

High levels of blood pressure and anxiety are common problems in the elderly (National Board of Health and Welfare, 2016) and might reflect an age related increased activity of the sympathetic nervous system and the hypothalamo–pituitary–adrenal (HPA)-axis. The reasons behind are complex; aging per se, physical inactivity and social deprivation including too little sensory stimulation may contribute. Pharmaceutical drugs are often given to the elderly, for example in order to reduce anxiety and high blood pressure. These pharmacological treatments might have side effects, such as an increased risk of unstable blood pressure, dizziness, and falls in older adults (National Board of Health and Welfare, 2016).

A possible non-pharmacological way to reduce such problems could be interaction with dogs. It appears as if dogs can induce stress-reducing and health promoting effects not only in humans they are familiar with and bonded to, but also in unfamiliar humans. For example, dogs trained to interact with strangers (therapy dogs), have been shown to reduce stress levels for example in healthcare professionals (Barker and Wolen, 2008), and to induce better Quality of Life in patients with Alzheimer’s disease and other forms of dementia (Nordgren and Engstrom, 2014; Swall et al., 2015). In addition, therapy dogs have been shown to increase social interactive behavior (Berry et al., 2012) and to reduce blood pressure and heart rate (Handlin et al., 2018) in residents of homes for the elderly.

Several senses are involved when humans interact with dogs. Eye contact with the dog, talking with and listening to the dog as well as caressing and stroking the dog, all constitute important components of the interaction. The important role of tactile interaction with the dog for stress reduction in humans has been demonstrated in several studies. Dog owners who pet their own dogs display decreased cortisol levels, blood pressure and heart rate (Odendaal and Meintjes, 2003; Handlin et al., 2011, 2012). Children who were allowed to pet a dog before performing a stressful task released less cortisol (as measured by salivary cortisol) than did children who were offered social support by an adult or who were allowed to pet a stuffed dog (Beetz et al., 2012). In fact, the more the children stroked the dog, the more their cortisol levels decreased indicating a quantitative relationship between the amount of stroking and stress reduction in the children (Beetz et al., 2012).

Results from animal studies support the existence of a link between sensory stimulation of the skin and stress reduction. Massage like stroking of the ventral side of rats is associated with lowering of blood pressure and heart rate (Lund et al., 1999; Holst et al., 2002) as well as by behavioral sedation (Uvnas-Moberg et al., 1996). In addition, activation of cutaneous nerves by low intensity stimuli induces powerful anti-stress effects such as lowering of blood pressure and heart rate, and also of cortisol and adrenaline levels (Kurosawa et al., 1982, 1995; Araki et al., 1984; Tsuchiya et al., 1991; Uvnas-Moberg et al., 1993). Taken together, these results indicate that stroking or other forms of tactile interaction with dogs induce stress reduction in the person who is performing the interaction by activation of cutaneous sensory nerves (Stock and Uvnas-Moberg, 1988).

Oxytocin is also released in response to interaction between humans and dogs (Odendaal and Meintjes, 2003; Miller et al., 2009; Handlin et al., 2011) as well as following stimulation of cutaneous sensory nerves in rats (Stock and Uvnas-Moberg, 1988), demonstrating a release of oxytocin from the paraventricular (PVN) and supraoptic (SON) nuclei into the circulation. Administration of oxytocin is linked to powerful anti-stress effects, it for examples decreases blood pressure and cortisol levels (Petersson et al., 1996, 1999; Uvnas-Moberg et al., 2019). Therefore, it is likely that oxytocin released from oxytocin containing nerves emanating from the PVN, and which project to areas in the brain that are involved in the regulation of autonomic nervous tone, mediates the anti-stress effects induced by stroking or other types of stimulation of sensory nerves from the skin (Uvnas-Moberg et al., 2019).

In a previous article based on the same material as in the present article, we were able to demonstrate short and long-term lowering of blood pressure and heart rate in elderly residents of a nursing home in response to interaction with a dog (Handlin et al., 2018). The aim of the present article was to investigate whether visits by a therapy dog may also influence fingertip temperature and cortisol levels in residents at nursing homes for the elderly. Skin temperature can be used as an indirect marker of stress levels, since blood vessels in the skin constrict in response to increased sympathetic nervous tone and consequently a high sympathetic tone will be associated with low skin temperature (Vinkers et al., 2013). Cortisol levels in saliva were also measured as an expression of the activity in the HPA-axis that is closely related to sympathetic nervous tone.

## Materials and Methods

### Participants

This study included two groups, the dog group and the control group. The dog group received repeated visits by the researchers and for half of the study (weeks 3–6) also by a visiting dog and its handler. It consisted of 15 participants; 13 women (mean age: 89 years) and 2 men (mean age: 80 years) recruited from three nursing homes for the elderly (five participants from each home). The control group received repeated visits by the researchers only. It also consisted of 15 participants, 11 women (mean age: 83 years) and 4 men (mean age: 84 years) recruited from three additional nursing homes for the elderly (five participants from each home). Due to long-term illness and/or hospital stay 2 women in the dog group and 2 men and 2 women in the control group only participated occasionally (dog group: *n* = 13, control group: period 1 *n* = 15, Period 2 *n* = 13, Period 3 *n* = 11). To be included in the study the participants had to fulfill the following criteria; be Swedish speaking and able to take part in a conversation, be able to make their own decisions and understand information and instructions. In addition, they should not have been diagnosed with dementia. All six nursing homes that were visited were located in the south west part of Sweden and none of them had any previous experience of visiting dogs.

### Ethical Consent

The experimental procedure was approved by the local ethics committee in Gothenburg, Sweden (dog study: 669-10, control study: 553-12). Before the experiment started, the participants, as well as their relatives, were informed about the study and given the opportunity to ask questions. They were also informed that they could end their participation in the study at any time without this affecting the care they received. The participant or a relative (in case the participant was unable to write) signed a written consent for participation in the study.

### Visiting Dog

The visiting dog was a privately owned 2-year-old female Labradoodle certified therapy dog, trained to work with people in elderly care. Both the dog and the dog handler had been trained for one year at “Vårdhundskolan” in Uppsala, Sweden. The experimental procedure was approved by the animal ethics committee in Uppsala, Sweden (283/10) and the use of a privately owned visiting dog was approved by the Swedish board of agriculture (D31-12610/10). The same visiting dog and its handler visited all three nursing homes.

### Study Design

In the dog group the elderly participated in the study during eight consecutive weeks. The researchers visited the nursing homes twice weekly during all 8 weeks whereas the Animal Assisted Activity (IAHAIO, 2018). i.e., the visiting dog and her handler, took place during weeks 3–6 only. Each visit lasted for 60 min. This design allowed the participants to get used to the testing situation before introducing the dog and her handler. It also allowed exploration of effects of interaction between the elderly and the dog in the dog group during visits (short term effects) and in response to the dog visits (long term perspective).

In the control group the elderly participated in the study during six consecutive weeks with two visits each week by the two researchers only and each visit lasted for 40 min. The reason for the shorter study period as well as shorter visits for the control group were problems in keeping the elderly residents motivated to continue the study and stay awake during the visits when no dog participated.

For analysis the study period was divided into periods; Period 1 (week 1–2), Period 2 (week 3–4), Period 3 (week 5–6) and Period 4 (week 7–8, only applicable for the dog group).

#### Experimental Setting

The five participants from each nursing home met the research team together as a group in the living room of the nursing homes. The temperature was normal indoor temperature (20–22°C). During the visits the group of participants sat in their own wheelchairs or in other suitable chairs, next to each other in a semi-circle. The only people present in the living room during the visits were the participants and the researchers who performed the data collection. The visiting dog and her handler were present for the dog group during weeks 3–6 (period 2–3) and during these weeks there was a water bowl and some toys for the dog in the living room.

During the visits the researchers did not initiate conversations with the participants but did answer questions posed by them. The researches spent the time between measurements sitting in their chairs without interacting with the residents.

#### Dog Visits in the Dog Group

The visiting dog always started each visit by greeting each participant; either by placing her head in the participant’s lap or letting the participant stroke her. The participants talked to and/or engaged in physical activity with the dog, including stroking the dog and playing with the dog by throwing a ball or another toy that the dog fetched. The participants also rewarded the dog by giving her treats when she acted upon her handler’s commands (e.g., sit, lie down, etc.). All participants interacted with the dog according to their individual capabilities. In case physical issues prevented the participants from bending or reaching the dog in order to touch it, a chair for the dog to sit on was placed next to the participant, allowing all participants to have physical contact with the dog. The handler made sure that the dog spent an equal amount of time with each participant during each visit of 60 min.

### Collection of Data

Fingertip temperature was measured with a Digital-Laser Thermometer (Esska de GmbH, Hamburg, Germany). Saliva to be used for cortisol analysis was collected using the SalivaBio Children’s Swab (SCS), and cortisol levels were analyzed with a commercial kit according to the manufacturer’s instructions (Salimetrics Europe Ltd., United Kingdom).

**FIGURE 1:**
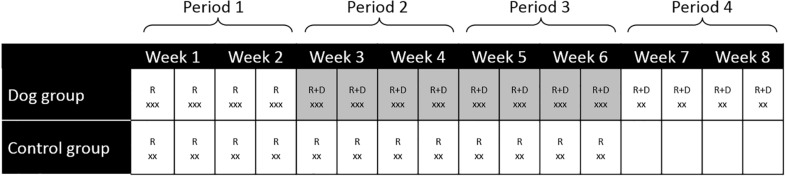
Summary of the study design and data collection for the dog group and the control group. R, researchers visited the participants, D, dog and dog handler visited the participants, x, number of measurements of fingertip temperature and collection of saliva for cortisol determinations. For the dog group the measurements were performed at 0, 20 and 60 min. For the control group the measurements were performed at 0 and 20 min. Highlighted weeks indicate meeting the visiting dog. Only two samples were collected for the control group and during week 7–8 (visit 13–16) in the dog group.

Measurements of finger temperature and collection of saliva was performed during each visit; at the start (0 min), after 20 min and at the end of the experiment (60 min) for the dog group and at the start (0 min) and after 20 min for the control group (Figure 1).

#### Statistical Analysis

Statistical calculations were performed using the statistical program SAS (Statistical Analysis System Inc, Cary, United States, version 9.4). In order to analyze the studies, mean values of the experimental variables were calculated per two weeks (mean value of data from four data collections) [periods 1 (weeks 1–2), 2 (weeks 3–4), 3 (weeks 5–6), and 4 (weeks 7–8)]. Mean values for periods with standard error of the means (SE) were created both for the values obtained at 0, 20, and 60 min combined, as well as separately.

Analyses were performed with two different statistical models in the mixed linear analysis procedure (proc mixed): The first model was used to test if there was a difference between the different experimental conditions (dog vs. control group), period (1, 2, or 3), sampling time (0 or 20 min) and interaction between experiment and period (indicating different effect patterns).

The second model was used to analyze only the dog group in more detail. This model also included values obtained at sampling at 60 min and period 4. This model tested if there was an effect of period (1, 2, 3, or 4), sampling time (0, 20, and 60 min) as well as of the interaction between period and sampling time.

Both models used an autoregressive covariance structure and Kenward–Roger for fixed effects SE method and degrees of freedom method. Type 3 tests of fixed effects were used to get *p*-values, *F*-values and degrees of freedoms (Num DF, Den DF). Random variable was id of the elderly and visit within experiment, and repeated variable was visit/subject. Post hoc test was done with paired *t*-tests where Tukey–Kramer adjusted *p*-values were used to compensate for multiple comparisons.

## Results

### Fingertip Temperature Over Time in the Dog Group and the Control Group

**FIGURE 2:**
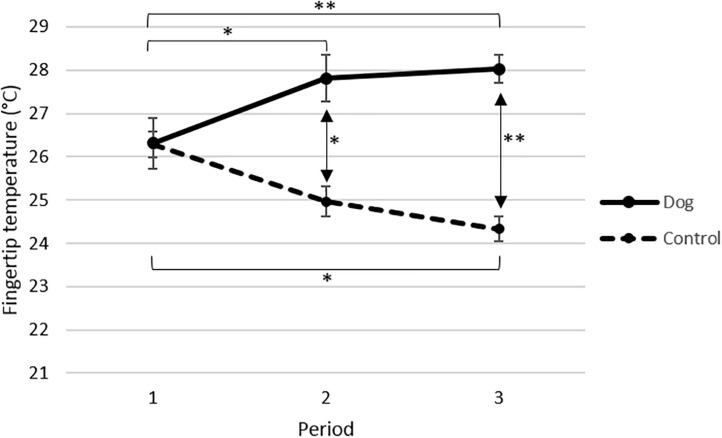
Mean fingertip temperature °C (±SE) for elderly before (period 1) and in connection with interacting with a visiting dog and its handler (periods 2 and 3) in the dog group (*n* = 13), and for elderly not interacting with a visiting dog during the corresponding time points in the control group (P1 *n* = 15, P2 *n* = 13, P3 *n* = 11). All data are based on mean values from sampling times 0 and 20 min. Data from each period are based on four separate visits. *p*-Values are adjusted from Tukeys post hoc test. ^∗∗^*p* < 0.01, ^∗^*p* < 0.05.

The fingertip temperature increased significantly in the dog group from period 1 to period 2 (*p* < 0.05) and from period 1 to period 3 (*p* < 0.01), but there was no significant increase from period 2 to period 3 (Figure 2). In contrast, the fingertip temperature decreased in the control group and the decrease was significant from period 1 to period 3 (*p* < 0.05) but not between period 1 to period 2 nor period 2 to period 3 (Figure 2). Taken together these data demonstrate that fingertip temperature rose in the dog group, when the elderly were visited by the dog and its handler, and that no such effect was observed in the absence of visits by the dog and its handler in the control group.

In support of the different effects observed in the dog group and the control group the statistical analysis showed a significant interaction between the treatments (dog or control) and the periods (*p* < 0.001, F2, 15.6 = 14.84).

When the values obtained during periods 1, 2 and 3 in the dog group and the control group were compared no differences in fingertip temperature were observed in period 1, i.e., at the start of the experiment (Figure 2). However, both during periods 2 (*p* < 0.05) and 3 (*p* < 0.001), the elderly in the dog group had significantly higher fingertip temperature than the elderly in the control group (Figure 2). The statistical analysis showed that the fingertip temperature was overall significantly higher in the dog group than in the control group (*p* < 0.0001, F1, 22.8 = 23.34).

### Fingertip Temperature Within the Dog Group

**FIGURE 3:**
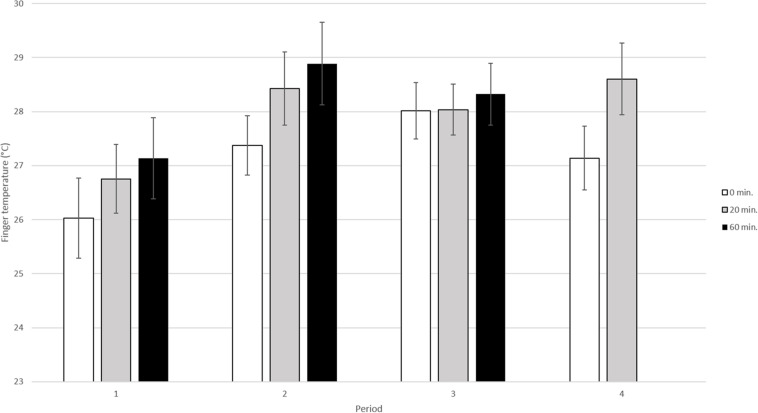
Mean fingertip temperature in °C (±SE) in the elderly obtained during the four different periods. The dog and its handler were present during period 2 and 3 but not during period 1 and 4. During each visit fingertip temperature was recorded three times; at 0 min, i.e., before the dog entered, at 20 min, when the dog was present and at 60 min, i.e., when the dog had left (*n* = 13 elderly sampled four times per period).

In this analysis data from all 4 periods and for the three sampling times (0, 20, and 60 min) were included (Figure 3). The results of the analysis showed that both period and sampling time significantly influenced fingertip temperature [(*p* < 0.01, F3, 150 = 4.29) and (*p* < 0.01, F2, 95.7 = 6.25) respectively].

More specifically, when comparing the fingertip temperature during the four different periods, the fingertip temperature was shown to be significantly lower in period 1 than in period 2 (adjusted *p* < 0.05), period 3 (adjusted *p* < 0.05) and period 4 (adjusted *p* < 0.05). However, there were no significant differences between the other periods [period 2 vs. period 3 (adjusted *p* > 0.1), period 2 vs. period 4 (adjusted *p* > 0.1), period 3 vs. period 4 (adjusted *p* > 0.1)].

When comparing the fingertip temperature for the different sampling times (0, 20, and 60 min) within all four periods, fingertip temperature was significantly lower at 0 min than at 20 min (adjusted *p* < 0.05) and 60 min (adjusted *p* < 0.01). There were no significant differences between 20 and 60 min (adjusted p > 0.1).

Comparisons of fingertip temperature between the three observations (0, 20, and 60 min) within each period (1–4) did not show any significant difference (adjusted *p* > 0.1 for all pairwise comparisons). Further, there were no significant interaction between period and sampling time (*p* > 0.1, F5, 136 = 0.25).

### Cortisol Levels

Only a subgroup of the saliva samples collected from the elderly (51 saliva samples in the dog group and 142 in the control group) were successfully analyzed for cortisol, probably as a consequence of low saliva production in the elderly. As only some of the saliva samples were analyzed (more samples from the control group than for the dog group), no statistical calculations could be performed. Mean values and standard errors for each sampling time and period are shown in Table 1.

**TABLE 1:** Mean values (±SE) of saliva cortisol in elderly residents in samples collected at 0 and 20 min during periods 1–4 in the dog group and 1–3 in the control group.

**Period**	**Sampling time**	***N***	**Dog group**	***N***	**Control group**
1	0	2	0.765 (0.430)	15	0.296 (0.020)
1	20	2	0.312 (0.0489)	15	0.272 (0.026)
1	60	3	0.570 (0.342)	–	–
2	0	3	0.388 (0.133)	10	0.290 (0.030)
2	20	7	0.226 (0.043)	11	0.291 (0.031)
2	60	5	0.438 (0.297)	–	–
3	0	3	0.190 (0.040)	8	0.260 (0.052)
3	20	2	0.225 (0.045)	6	0.234 (0.049)
3	60	3	0.320 (0.165)	–	–
4	0	4	0.251 (0.050)	–	–
4	20	3	0.347 (0.253)	–	–

## Discussion

In the present study fingertip temperature in connection with interaction with a visiting dog was investigated in elderly people living in nursing homes. Elderly who interacted with a visiting dog and its handler displayed significantly increased fingertip temperature over time, i.e., temperature increased during the 4 week period of dog visits as well as within the single visits in response to interaction with the dog. In a control situation, i.e., when elderly received visits by the researchers only without the dog and its handler, no such rise in fingertip temperature was seen.

Rapid changes in skin temperature in response to moderate changes in temperature are mediated by changes in sympathetic nervous tone. When sympathetic nervous tone is high, blood vessels in the skin contract, which will be associated by reduced skin blood flow and lowering of skin temperature. In contrast when sympathetic nervous tone is decreased cutaneous blood vessels dilate and skin-temperature increases (Vinkers et al., 2013). Therefore, it is suggested that the increased skin temperature induced in the elderly by interacting with a visiting dog and its handler is mediated by a decrease in sympathetic nervous tone in the blood vessels of the skin.

In order to investigate whether the activity in the HPA-axis also decreased, an attempt to measure cortisol levels was performed. Saliva was taken from the elderly during the experiments and cortisol levels were analyzed. Unfortunately, the volume of a majority of the saliva samples was too small to allow cortisol determination, probably as a consequence of low saliva production in the elderly. However, the samples that were analyzed do not contradict that cortisol levels also fall in response to interaction with dogs, although the number of samples is too low to make any significant statements.

In a previous article we reported that both blood pressure and heart rate fell within the same population of elderly residents of nursing homes in response to interaction with the visiting dog (Handlin et al., 2018). Also other studies indicate that blood pressure and heart rate decrease in response to physical interaction with a dog. In addition, cortisol levels decrease in response to stroking of dogs (Odendaal and Meintjes, 2003; Handlin et al., 2011). Taken together, the data show that physical interaction with dogs give rise to immediate and sustained anti-stress effects.

In a previous study on dog owners, stroking of their own dog was shown to increase circulating levels of oxytocin in addition to a decrease of cortisol levels (Handlin et al., 2011). Similar data was also obtained by Odendaal and Meintjes (2003), who showed that oxytocin levels rose and that cortisol, blood pressure and heart rate fell in response to stroking of a dog (Odendaal and Meintjes, 2003). Given the potent anti-stress effects of oxytocin; its ability to decrease the activity of the HPA-axis and of the sympathetic nervous system via actions in the brain, it is likely that oxytocin released within the brain, as a response to the interaction with the therapy dog, causes the anti-stress effects observed.

The oxytocin release and consequent anti-stress effects induced by interaction with the dog, is probably caused by sensory stimuli such as touch, light pressure and stroking known to cause oxytocin release (Uvnas-Moberg et al., 2014). In support of an important role for stroking, cortisol levels were dose dependently decreased when young boys exposed to a stress test were allowed to stroke a dog (Beetz et al., 2012).

Physiological and psychological effects of stroking have been shown to be linked to an activation of several types of cutaneous nerves in humans. Stimulation of a subgroup of the thin unmyelinated C-fibers, the Ct – fiber afferents, has been shown to be linked to activation of certain areas in the insular cortex and to the experience of wellbeing (Loken et al., 2009). Thicker myelinated nerve fibers are activated in response to light/moderate pressure with or without stroking and activation of such fibers is linked to anti stress effects (Uvnas-Moberg et al., 2019). Data from animal experiments also demonstrate powerful anti-stress effects in response to activation of sensory nerves from the skin, either by non-noxious stimulation of cutaneous nerves in anesthetized animals or in response to massage-like stroking of the ventral side of rats (Uvnas-Moberg et al., 1996; Sato et al., 1997; Lund et al., 1999).

All these data suggest that stimulation of cutaneous nerves is an important mechanism by which interaction with dogs decreases stress levels in humans. It is of course important to remember that touch and stroking are not the only mechanism by which interaction with dogs influences the elderly. Seeing the dogs, listening to them, smelling them, and playing with them probably also contribute to the oxytocin release and associated anti-stress effects. The role of the person handling the dog should also be considered. In this study the handler guided the interaction between the dog and the participant, which included her both touching and talking to the participants. Therefore it is not possible to distinguish between the effects caused by the dog and the effects caused by the handler and it might be more appropriate to look at the effects observed in the dog group as a result of the dog/handler team. It is also important to note that the entire study situation in itself can have an effect since it is a novelty compared to the participant’s regular schedule. The results from the control group does, however, indicate that it was the dog visits that had the most pronounced effect since meeting the researchers only did not generate any increase in fingertip temperature. Further studies are needed to dissociate and to describe in more detail the role of the individual sensory components responsible for the anti-stress effects observed following interaction between the elderly and a visiting dog.

Skin-temperature as a marker for anti-stress effects, more specifically for a decreased sympathetic nervous tone, has been used in several other studies. The decrease in fingertip temperature observed in children experiencing stress when visiting a dentist, was less expressed in the presence of a dog, indicating that the dog buffered the stress response in this situation (Havener et al., 2001). Increased fingertip temperature coincides with a cluster of anti-stress effects, such as lowering of heart rate and blood pressure, and it is a physiological indicator of relaxation. Application of several methods aimed at stress reduction and relaxation, (for example, eye movement desensitization reprocessing (EMDR) and many others) are linked to an increased fingertip temperature (Wilson et al., 1996; Schlader et al., 2010). It seems that using changes in fingertip temperature represents not only a sensitive technique for measurement of anti-stress effects, it is also noninvasive and easy to perform. Therefore, recording of fingertip skin temperature should be used more often to demonstrate changes in stress levels.

The results presented in this study is based on a relative small number of participants and unfortunately as it was not possible to randomize the elderly participants to the different groups. The study design and statistical approach does, however, make up for this to some point. The result presented here are based on substantial amount of measurements since each value used for statistical calculations was based on 24 recordings, which allowed us to see significant changes and differences over time within the two groups and also between the two groups. It would also been desirable to visit both groups in parallel but for logistical reasons this was not possible since we wanted the nursing homes to be as similar as possible and within the same region.

The data presented in this study provides further support for decreased stress levels following interaction between humans and dogs, in this case between a visiting dog and elderly people living in a nursing home. Exposure to the visiting dog resulted in an increase in fingertip temperature, an expression of decreased sympathetic nervous tone. The data may also suggest that a sustained increase in skin temperature, reflecting a more long term reduction of stress levels, may be induced in the elderly after repetitive exposure to the visiting dog. Such a long-term effect was previously shown for heart rate and blood pressure (Handlin et al., 2018). Sustained anti stress effects in response to interaction between humans and animals should be of particular importance for health promotion.

## Conclusion

In the present study interaction between elderly residents and a visiting dog resulted in increased fingertip temperature, probably reflecting a decrease in the activity of the sympathetic nervous system and consequently a decrease in stress levels.

## Data Availability Statement

The datasets presented in this article are not readily available because the application and the written consent forms approved by the local ethics committee in Gothenburg, Sweden, states that the data will only be available to the researchers within the project. Requests to access the datasets should be directed to linda.handlin@his.

## Ethics Statement

The studies involving human participants were reviewed and approved by The local ethics committee in Gothenburg, Sweden. The patients/participants provided their written informed consent to participate in this study. The animal study was reviewed and approved by The animal ethics committee in Uppsala, Sweden and the Swedish board of agriculture.

## Author Contributions

AN contributed with the data collection, methodological discussions, data interpretation, and manuscript writing. LH contributed with the data collection, statistical analysis, methodological discussions, data interpretation, manuscript writing, and critical editing. LL and MP contributed with the methodological discussions, data interpretation, manuscript writing, and critical editing. KU-M was main responsible for the project and contributed with the methodological discussions, data interpretation, manuscript writing, and critical editing. All authors contributed to the article and approved the submitted version.

## Conflict of Interest

The authors declare that the research was conducted in the absence of any commercial or financial relationships that could be construed as a potential conflict of interest.
